# Emirati Adults Have a Higher Overall Knowledge on Vitamin D Compared to Tourists

**DOI:** 10.3389/fpsyg.2020.01022

**Published:** 2020-06-03

**Authors:** Ahlam Saleh, Jawaher Saeed Alhadhrami, Maei Saeed Al Ramahi, Halima Ali Albloushi, Rafiq Hijazi, Myriam Abboud, Dimitrios Papandreou

**Affiliations:** ^1^Department of Health Sciences, College of Natural and Health Sciences, Zayed University Abu Dhabi, Abu Dhabi, United Arab Emirates; ^2^Department of Math, College of Natural and Health Sciences, Zayed University Abu Dhabi, Abu Dhabi, United Arab Emirates

**Keywords:** knowledge, vitamin D, calcium, physical activity, nutrition, Emiratis, tourists

## Abstract

**Objectives:**

In the last decade, vitamin D deficiency has become a major global issue. One of the main functions of vitamin D is the proper absorption of calcium in the gastrointestinal track. Optimal vitamin D levels are mandatory for adequate calcium absorption and bone health. The purpose of this study was to assess the level of knowledge of vitamin D, calcium, and physical activity among Emirati and tourist adults in Abu Dhabi.

**Methods:**

This is a cross-sectional study that took place in three different malls in Abu Dhabi and included Emirati and tourist adults. Participants were asked to fill in a questionnaire consisting of 32 questions. These included questions on vitamin D, calcium, supplement, and physical activity knowledge. Another section of the questionnaire included general information on age, sex, education, weight, and height. The collected data were statistically analyzed using descriptive statistics using IBM SPSS statistics for Windows version 26.0 (SPSS Inc., Chicago, IL, United States). Statistical significance was set at *P* < 0.05.

**Results:**

Out of 147 adults, 113 were females and 34 males. The mean age, height, and weight were 27.9 ± 8.6 years, 162.7 ± 10.4 cm, and 66.5 ± 19.5 kg, respectively. Emiratis had statistically significant higher basic knowledge on vitamin D compared to tourists (44.9 vs 27.1%), respectively. More than 66% of the whole sample was aware that vitamin D deficiency can affect muscle strength, as well as that calcium may affect osteoporosis. In a multiple regression model to analyze the possible effects of other factors to knowledge, it was found that only age (Beta: 0.045, *P* < 0.014) and nationality (Beta: 0.750, *P* < 0.018) were independently and significantly associated to vitamin D.

**Conclusion:**

Emirati participants showed a higher overall vitamin D knowledge than their tourist counterparts. Both groups had low/medium level of knowledge when it comes to physical activity and calcium and vitamin D supplements.

## Introduction

Vitamin D and calcium levels have been a major concern in the last 20 years, and many studies have been conducted to analyze how diet and supplements may provide a beneficial effect especially to those people who are deficient (Holick and Chen, 2008; Alemu and Varnam, 2012). Vitamin D deficiency in both children and adults poses a major global health issue (Wahl et al., 2012).

Vitamin D mainly contributes to the regulation of calcium and phosphate metabolism and the maintenance of a healthy skeleton. Humans synthesize vitamin D primarily from sun exposure; however, other sources of vitamin D are provided through intake of selected foods and nutritional supplements (Charoenngam et al., 2019). Deficiency of vitamin D is very prevalent in the Middle East region, and it has recently reached 81% among various age groups (Mithal et al., 2009; Holick, 2017).

There are two forms of vitamin D: vitamin D_3_ and vitamin D_2_. Both precursors come from diet and sunlight and are finally converted in the kidney to form 1, 25-dihydroxyvitamin D (calcitriol), which is the active form (Zhang and Naughton, 2010). The Endocrine Society’s Clinical Practice Guideline defines vitamin D deficiency, insufficiency, and sufficiency as serum concentrations of 25(OH)D of <20, 21–29, and 30–100 ng/ml, respectively.

One of the major functions of vitamin D is the absorption of calcium in the gastrointestinal track. Optimal vitamin D levels are mandatory for adequate calcium absorption. It has been observed that the body can only absorb 10–15% if vitamin D status is not adequate (Khazai et al., 2008).

In addition, physical activity (PA) is an important factor in health promotion and disease prevention. Lack of PA has been shown to contribute to the development of many chronic diseases such as diabetes type 2 and cardiovascular disease. Consequently, PA guidelines have been developed to give people knowledge about what is, in general, a minimum amount of PA to improve health. The recommendations concluded that adults should exercise for 30 m on most days of the week (Vaara et al., 2019). The lack of knowledge on vitamin D, calcium, and PA could be a potential risk factor for the development of non-communicable diseases such as osteoporosis, cardiovascular disease, and cancer. Data from United Kingdom, Saudi Arabia, United Arab Emirates, and Kuwait report mixed results on the lack of awareness and knowledge on vitamin D (Alemu and Varnam, 2012; Al-Mutairi et al., 2012; Salmanpour et al., 2016; Zhou et al., 2016; Alamoudi et al., 2019; Almuqati et al., 2019).

The purpose of this study was to assess the overall knowledge on vitamin D, calcium, supplements, and PA among Emirati and tourist adults in Abu Dhabi.

## Materials and Methods

This is a cross-sectional study that took place in three different malls in Abu Dhabi and included Emirati and tourist adults. Initially, it was scheduled to invite 200 adults to participate, but due to the coronavirus disease, we succeeded to collect only 147 subjects (98 Emiratis and 49 tourists).

The inclusion criteria were age >18 years old, not taking any medication or vitamin supplements, and not having a medical condition such as cardiovascular disease, diabetes, or osteoporosis. The researchers were waiting outside of malls and were randomly approaching the subjects. First some basic questions were asked to make sure the selected participants were qualified to participate in the study; then, they were administered with the questionnaire. The questionnaire included 32 basic knowledge questions on vitamin D, calcium, vitamin D and calcium supplements, and PA knowledge. Another section of the questionnaire included general information about the characteristics of the participants such as age, sex, education, weight, and height.

The level of knowledge was defined as Low/Medium/High, where Low was <40% correct responses to all questions, Medium from 41 to 70%, and High >70%. The study was approved by the ethical committee of Zayed University, and all participants signed a consent form.

### Statistical Analysis

The IBM SPSS Statistics (version 26) was used to enter, prepare, and analyze the collected data. Descriptive statistics including the means and standard deviations were reported for numerical data, and the frequencies and percentages were used to summarize the categorical data. The Chi-square test of homogeneity was used to test for differences in individual knowledge questions between Emiratis and tourists, while the independent two-sample t-test was employed to examine differences in the knowledge components between Emiratis and tourists. Statistical significance was set at *P* < 0.05.

## Results

Table 1 presents the characteristics of the sample. Out of 147 adults, 113 were females and 34 males. The mean age, height, and weight were 27.9 ± 8.6 years, 162.7 ± 10.4 cm, and 66.5 ± 19.5 kg, respectively. Out of the total sample, 67.3% were Emirati and 32.7% were tourists, while 39.1% were overweight and obese.

**TABLE 1:** Characteristics of study participants according to gender (*n* = 147).

	**Male (*n* = 34)**		**Female (*n* = 113)**		**Total (*n* = 147)**	
	**Frequency**	**Percentage**	**Frequency**	**Percentage**	**Frequency**	**Percentage**
Age (years)	32.2 ± 9.8		26.6 ± 7.8		27.9 ± 8.6	
Height (cm)	172.1 ± 9.8		159.8 ± 8.9		162.7 ± 10.4	
Weight (KG)	80.6 ± 24.2		62.2 ± 15.7		66.5 ± 19.5	
**BMI**						
Underweight	1	2.9	13	11.5	14	9.5
Normal	14	41.2	60	53.1	74	50.3
Overweight	11	32.4	20	17.7	31	21.1
Obese	8	23.5	20	17.7	28	19.0
**Nationality**						
Emirati	14	41.2	85	75.2	99	67.3
Tourists	20	58.8	28	24.8	48	32.7
**Education level**						
High School or less	9	26.5	27	23.9	36	24.5
Diploma	3	8.8	20	17.7	23	15.6
Bachelor	15	44.1	55	48.7	70	47.6
Postgraduate	7	20.6	11	9.7	18	12.2
**Taking medication**						
Yes	6	17.6	18	15.9	24	16.3
No	28	82.4	95	84.1	123	83.7

*Data expressed as mean ± standard deviation.*

Table 2 compares the knowledge of Emiratis and tourists in all four categories. Emiratis had statistically significantly higher basic knowledge on vitamin D compared to tourists (44.9 vs 27.1%). Both Emirati and tourists had low/medium knowledge on PA and supplements.

**TABLE 2:** Comparison of knowledge level between Emirati and tourists.

**Knowledge component**	**Nationality**	**Knowledge level**		
		**Low (%)**	**Medium (%)**	**High (%)**
Vitamin D knowledge	Emirati	4.1	51.0	44.9
	Tourists	8.3	64.6	27.1
	Total	5.5	55.5	39.0
Calcium knowledge	Emirati	6.1	60.6	33.3
	Tourists	12.5	62.5	25.0
	Total	8.2	61.2	30.6
Physical activity knowledge	Emirati	30.3	59.6	10.1
	Tourists	25.0	47.9	27.1
	Total	28.6	55.8	15.6
Vitamin D and calcium supplements knowledge	Emirati	41.4	53.5	5.1
	Tourists	43.8	52.1	4.2
	Total	42.2	53.1	4.8

*Low (<40%), Medium (41–70%), High (>70%).*

The individual’s statistically significant responses on knowledge questions are presented in Table 3. More than 66% of the whole sample were aware that vitamin D deficiency can affect muscle strength, as well as that calcium may affect osteoporosis. Emirati had higher knowledge in six out of nine questions compared to tourist ones.

**TABLE 3:** Statistically significant responses of the sample to individual questions.

	**Tourists (%)**	**Emirati (%)**	***P*-value**
Q1: Can pancreatitis effect vitamin D absorption?	45.8	67.7	0.011*
Q2: Do you think that the deficiency of vitamin D can lead to baldness?	58.3	28.6	0.001**
Q3: Do you think that the deficiency of vitamin D can decrease muscle strength?	79.2	91.9	0.027*
Q4: Do you think that vitamin D deficiency can lower bone density?	50.0	77.8	0.001**
Q5: Does alcohol have an effect on vitamin D absorption?	68.8	85.9	0.015*
Q6: Do you think that animal sources (meats/egg) have more vitamin D than plant sources?	50.0	69.7	0.02*
Q7: Does dietary calcium affect osteoporosis?	81.3	96.0	0.003**
Q8: Are there any harmful effects if one takes more than 2,000 mg of calcium?	41.7	15.2	0.001**
Q9: Are vitamin D supplements safe, if you are not vitamin D deficient?	68.8	51.5	0.048*

*Statistically significant at *P* < 0.05. **Statistically significant at *P* < 0.01.

Table 4 shows the comparison of overall score of knowledge among Emirati and tourist sample. Emirati had statistically significantly higher knowledge on vitamin D compared to that of tourists. No significant differences were found on the other three parameters when comparing the two groups.

**TABLE 4:** Comparison of overall score of knowledge among Emirati and tourist sample.

**Knowledge component**	**Nationality**	***n***	**Mean**	**Standard deviation**	***t***	***P*-value**
Vitamin D knowledge	Emirati	98	6.92	1.71	2.74	0.007*
	Expatriate	48	6.06	1.88		
Calcium knowledge	Emirati	99	6.23	1.33	1.89	0.060
	Expatriate	48	5.76	1.56		
Physical activity knowledge	Emirati	99	5.32	1.87	–1.36	0.176
	Expatriate	48	5.80	2.25		
Vitamin D and calcium supplements knowledge	Emirati	99	4.51	1.89	–0.01	0.995
	Expatriate	48	4.51	1.68		

*Statistically significant set at *P* < 0.05.

Using a multiple regression model to analyze the possible effects of other factors to knowledge, it was found that only age (Beta: 0.045, *P* < 0.014) and nationality (Beta: 0.750, *P* < 0.018) were independently and significantly associated to vitamin D (Table 5).

**TABLE 5:** Regression analysis for factors affecting knowledge levels.

**Dependent variable**	**Vitamin D knowledge**			**Calcium knowledge**			**Physical knowledge**			**Supplements knowledge**		
				
	**B**	**SE**	***P*-value**	**B**	**SE**	***P*-value**	**B**	**SE**	***P*-value**	**B**	**SE**	***P*-value**
Intercept	3.297	0.824	0.000	5.770	0.843	0.000	3.797	0.653	0.000	2.832	0.585	0.000
Age	0.045	0.018	0.014*	0.021	0.018	0.245	–0.004	0.014	0.779	0.009	0.013	0.502
BMI	0.024	0.022	0.281	–0.003	0.023	0.894	–0.003	0.018	0.855	–0.022	0.016	0.161
Gender	0.494	0.329	0.135	0.174	0.336	0.605	–0.281	0.261	0.282	–0.071	0.233	0.760
Taking medication	0.321	0.356	0.369	–0.087	0.364	0.811	–0.042	0.282	0.882	–0.154	0.253	0.544
Nationality	0.750	0.312	0.018*	0.523	0.319	0.104	–0.222	0.247	0.371	0.192	0.221	0.387
Diploma	–0.282	0.416	0.499	0.113	0.426	0.792	0.379	0.330	0.253	0.379	0.296	0.202
Bachelor	0.189	0.320	0.556	–0.059	0.327	0.857	–0.066	0.253	0.794	–0.004	0.227	0.985
Postgraduate	–1.198	0.484	0.014	–0.583	0.495	0.241	0.205	0.384	0.593	0.697	0.343	0.044

*F*	3.180			0.802			0.664			1.137		
*P*-value	0.002			0.602			0.723			0.342		
*R*^2^	24.2%			9.2%			9.6%			9.4%		

*Statistical significance set at *P* < 0.05.

## Discussion

The current study shows that Emiratis have a higher overall knowledge on vitamin D compared to that of tourists. Both groups showed low/medium knowledge on calcium, supplements, and PA. Many local and international countries have reported mixed results. The countries that showed low awareness on vitamin D may also explain why these countries have a high percentage of vitamin D deficient population. On the other hand, similar data from the same countries showed that their population had very high awareness on vitamin D levels, and their results are in agreement with our data too (Alemu and Varnam, 2012; Zhou et al., 2016; Almuqati et al., 2019).

In a United Kingdom study, almost 54% of the participants had low knowledge on the basic symptoms of vitamin D deficiency, while 34% did not get enough sunlight, and 11% did not have enough vitamin D from food sources (Alemu and Varnam, 2012). A Chinese study assessing the knowledge of 515 students about vitamin D concluded that the participants had low knowledge levels on vitamin D, and they should include vitamin-rich food sources in their diet (Zhou et al., 2016).

In a recent cross-sectional study in Saudi Arabia, 1,022 participants’ knowledge on vitamin D was evaluated and it was concluded that there is a very high level of inadequate knowledge of vitamin D that is also related to the educational levels of the population (Alamoudi et al., 2019).

More recently, a cross-sectional study was also conducted in Sharjah, United Arab Emirates. A total of 503 adults were selected from public places using a convenience sampling method. Vitamin D deficiency knowledge was very common in the majority of the adults (Salmanpour et al., 2016).

On the other hand, and in the neighboring country of Kuwait, investigations of the level of awareness, knowledge, and attitude of representative groups from the general population toward sun protection showed that the majority of the population had good knowledge toward sun protection and its relation to vitamin D; however, low knowledge on calcium was observed (Al-Mutairi et al., 2012).

Similar results were also observed in another cross-sectional study that included students from Saudi Arabia. The authors evaluated 501 medical students and found that most students were aware of the risks of unprotected sun exposure. Almost 71% of the students were familiar with the association of vitamin D level and sun exposure (Almuqati et al., 2019).

It is worthy to note that our study has many limitations: (a) It does not represent the population of Abu Dhabi, (b) the sample size was small, and (c) we did not have information whether the sample consumed any vitamin D or calcium supplements the last year. The last one could affect significantly the knowledge level. Nevertheless, our study provides some initial data about the knowledge level on vitamin D and the need for larger sample studies in the near future.

## Conclusion

The study highlights a high knowledge level among the Emirati participants on dietary vitamin D compared to tourists; however, a low/medium knowledge level of both groups on calcium, vitamin D and calcium supplements, and PA was observed. Future intervention studies on a large sample are needed to accurately investigate the level of awareness in order to determine the possible reasons for the low knowledge of calcium and PA.

## Data Availability Statement

The data used to support the findings of this study are available upon request from the corresponding author.

## Ethics Statement

The studies involving human participants were reviewed and approved by the Research Ethical Committee, Zayed University. The patients/participants provided their written informed consent to participate in this study.

## Author Contributions

RH and MA contributed to the conception, design, and statistical analysis of the study. AS, JA, MA, and HA collected the data and organized the database. DP contributed to the conception and design of the study and wrote the first draft of the manuscript. All authors contributed to manuscript revision and approval.

## Conflict of Interest

The authors declare that the research was conducted in the absence of any commercial or financial relationships that could be construed as a potential conflict of interest.
